# Home and Office Blood Pressure Monitoring in Renal Transplant Recipients

**DOI:** 10.1155/2012/702316

**Published:** 2012-04-17

**Authors:** Rebecca Sberro-Soussan, Marion Rabant, Renaud Snanoudj, Julien Zuber, Lynda Bererhi, Marie-France Mamzer, Christophe Legendre, Eric Thervet

**Affiliations:** ^1^Service de Transplantation Rénale Adulte, Hôpital Necker, AP-HP, 75015 Paris, France; ^2^Université Paris Descartes, 75006 Paris, France; ^3^Service de Néphrologie, Hopital Européen Georges Pompidou, AP-HP, 75015 Paris, France

## Abstract

*Background*. Arterial hypertension in renal transplant recipients (RTR) is associated with increased morbid mortality. In the general population, home blood pressure monitoring (HBPM) was found to be superior to office blood pressure (OBP) in identifying true hypertensive patients. The aim of this study was to investigate HBPM for the assessment of blood pressure profile in RTR. *Methodology and Principal Findings*. We included prospectively 87 stable RTR. Sitting OBP was measured during the outpatient clinic. HBPM was performed by measuring BP every morning and night for 4 days. The accepted limits for the OBP and HBPM, were respectively, 140/90 mmHg and 135/85 mmHg. Patients were classified as “normotensive,” “uncontrolled,” “white-coat hypertensive” and “masked hypertensive”, (OBP below the limit and HBPM above). During the study, 81 patients (55 males, age 48.5 ± 14 years) were available for analysis. The mean OBP and HBP were 138/83 ± 14/10 mmHg and 133/79 ± 14/8 mmHg; 29% of patients were uncontrolled, 28% normotensive, 21% white coat, and 21% masked hypertensive. Age, glycemia, and number of antihypertensive drugs were associated with hypertension. *Conclusion and Significance*. In RTR, HBPM is well accepted and better define BP profile since there is 42% discrepancy between OBPM and HBPM. Whether this discrepancy is associated with worst outcome in the long term remains to be demonstrated.

## 1. Introduction

Arterial hypertension is prevalent in renal transplant recipients (RTR) and is a powerful predictor of impaired graft and patient outcome [1]. Diagnosis of arterial hypertension has traditionally been based on measurements of blood pressure (BP) in the office or clinic. However, it is known for many years that BP, in most individuals, is higher in this setting than in home. It is now well recognized that out-of-office recordings of BP yield better prognostic information than those obtained in physician's offices [2–4]. A recent review has emphasized the advances of out-of-office recordings in patients with chronic kidney disease [5]. Home measurements can be obtained either by ambulatory BP monitoring or self-measurement of BP. Both these techniques allow detecting white-coat hypertension and masked hypertension. White-coat hypertension is defined as well-controlled home hypertension but poorly controlled clinic hypertension. Masked hypertension is the reverse phenomenon, poorly controlled BP at home, but normal in the clinic [6]. Furthermore, ambulatory BP monitoring gives more measurements and may identify the variability of hypertension which may be of importance [7, 8]. However, when BP is measured by several methods (nurse, home, ambulatory, and doctor), it has been showed that home measurements may be the most promising option, as they are the most acceptable method to patients and were preferred to other readings in the ambulatory monitoring. Ambulatory monitoring performs less well than other methods, largely owing to discomfort and disturbance of life and sleep [9, 10]. To clarify the level of agreement among these different blood pressure measures, a recent study compared and showed that under rigorously standardized conditions, clinic and home BP could be used as an alternative to awake ABP [11]. Even though some data exist regarding ambulatory BP monitoring in RTR, there is only few data available concerning home BP monitoring in this specific population [12].

## 2. Methods

### 2.1. Objectives

The aim of this study was to evaluate the acceptability of home BP monitoring, to define the prevalence of hypertension with this technique, and to describe the existence of white-coat hypertension and masked hypertension in RTR. We also investigated the risk factors of patients with specific BP profile.

### 2.2. Participants

Between February and October 2009, we included in this prospective observational study 87 stable RTR. The inclusion criteria were as follow. Patients had to have received a single renal transplant for more than one year and to enjoy a stable renal function during the past 6 months defined as less than 20% of variability of serum creatinine. Immunosuppressive treatment had to be stable during 6 months, especially regarding daily dose and blood concentrations of calcineurine inhibitors (CNI). The patient had to be within targeted CNI blood concentrations, that is, cyclosporine concentrations 2 hours after intake (C2) between 400 and 800 ng/mL; tacrolimus blood trough levels (C0) between 4 and 12 ng/mL. Antihypertensive treatments were monitored and had to be identical for the past 6 months. Patients were not included if systolic BP was lower than 100 mmHg and higher than 190 mmHg.

### 2.3. Description of Procedures

#### 2.3.1. Blood Pressure Measurements

Both office and home blood pressure was measured using a MICROLIFE BP A100 Plus device (Microlife). This automatic device generates a digital display both of systolic blood pressure (SBP) and of diastolic blood pressure (DBP). It has previously been validated and satisfied the criteria of the association for the advancement of medical instrumentation (British Hypertension Society). The device calibration was the same for home and office. The cuff was adapted to the circumference of the arm contra lateral to the arteriovenous fistula (AVF) in patients whose AVF was still functioning. The machine was calibrated to take three measurements.

Office blood pressure was first measured by the physician during the clinic. The patients were then asked to have their BP taken once again by a nurse who instructs them on how to perform home BP measurements. BP measurements and information was given by a unique person (CC). Both physician and nurse measurements were obtained while patients were seated, and after they had rested for more than 5 minutes.

The instructions given how to perform home blood BP were as follows. Subjects were asked to measure their blood pressure every morning within 1 h of waking and every night (we asked patients to wait about 10 to 12 hours between morning and evening measurements), while they were seated and after they had rested for more than 5 minutes at the time they had taken their blood pressure medication and to record on a specific paper sheet the results for 4 days. We asked patients to wait about 10 to 12 hours between morning and evening measurements. After this period, patients returned their device and the written results were compared to the one recorded in the memory of the device.

#### 2.3.2. Arterial Hypertension Definition

The home blood pressure of an individual was defined as the mean of all measurements obtained for that person. The mean number of home blood pressure measurements was 8 (at least 4 in the morning and 4 in the evening). There was not significant difference in measurement of HBP in morning and at evening. Thus, we considered that the average of the both measurement of HBP was valid.

We used for this study the following definition for controlled and uncontrolled hypertension. For the office measurements, the internationally accepted limit of 140/90 mmHg was adopted. For home BP measurement, we adopted the internationally accepted limit of 135/85 mmHg.

Patients were classified in four groups as (i) “controlled” when their BP was below the limit for each of the methods; (ii) “uncontrolled” when their BP was greater or equal to the limit for each of the methods; (iii) “white coat hypertension” when their BP was below the limit of control for HBPM and greater than or equal to the limit for OBPM; (iv) “masked hypertension” when their BP was below the limit for the OBPM and greater than equal to the limit of control for the HBPM.

### 2.4. Ethics

The study obtained ethics approval from the ethics committee of the hospital named *“CPP (Comité de Protection des Personnes)”*. All patients gave informed written consent to enter the study.

### 2.5. Statistical Methods

Statistical analyses were performed with the SAS JMP software version 5.0.1 for MacIntosh (SAS Institute, Inc., Cary, NC). Characteristics of patients at the time of the study were described using means ± standard deviation or frequencies and compared with the chi-square test, the Fisher's exact test, and analysis of variance, as appropriate. Univariate and multivariate logistic regression were used to determine the odds ratio and confidence intervals. Multivariate analysis was performed after adjustment for variables identified as significant with *P* < 0.2 in univariate analysis.

A two-sided *P* value of 0.05 was considered to be statistically significant. Furthermore, we studied the relation between the GFR and measurement error by applying the method as proposed by Bland and Altman [13]. We assessed the bias as well as the 95% limits of agreement.

## 3. Results

### 3.1. Demographic Characteristics

We included 87 patients in this study. Of note, only 81 patients were available for analysis: 3 patients did not bring back the device; 3 patients did not perform home BP measurements as indicated. No patient had complains regarding the acceptability of BP measurements. Demographic characteristics of the patients are described in Table 1. Since we included stable patients with no limit regarding the delay after transplantation, the median delay was 3.08 years ranging between 1 and 31 years. At inclusion, the mean serum creatinine was 137 ± 39 *μ*mol/L. The immunosuppressive regimens were variable with 88% of patients receiving a CNI as part of the treatment. With regards to the number of antihypertensive at the time of the study, the mean number was 1.5 ± 1, with 16 patients receiving no therapy, 24 patients were taking one medication, 28 patients were on two medications, 11 patients were receiving 3 medications, and 2 patients 4 medications. Among the different antihypertensive medications, 62.8% of treated patients received beta-blockers, whereas angiotensin-converting enzyme inhibitors or angiotensin-2-receptor antagonists were used in 53% and calcium channel blockers in 28.2%. New onset diabetes after transplantation (NODAT) was defined by an HbA1c value higher than 6.5% or ongoing treatment for diabetes. In this population, 4 patients only were receiving antidiabetic treatment consisting in insulin (*n* = 1) or oral agents (*n* = 3).

Concerning the other cardiovascular risk factors, 56 patients (69.14%) were treated with lipid-lowering agents. Among them, all received statins and 6 Ezetimib either alone or in combination with statins. 

### 3.2. Blood Pressure

We found no discrepancy between the reported BP and reading values within the memory device. We had no phantom recordings. The mean systolic and diastolic BP values for physician were, respectively, 139 ± 14 mmHg and 83 ± 10 mmHg. The respective values were 134 ± 15 mmHg and 82 ± 9 mmHg for nurse BP. These measurements between nurse and physicians were concordant in 73% of patients (with 29% of hypertensive and 44% of normotensive patients). Of note, 19% of the patients were hypertensive with the medical doctor, although they were only 8% with the nurse.

**Figure 1:**
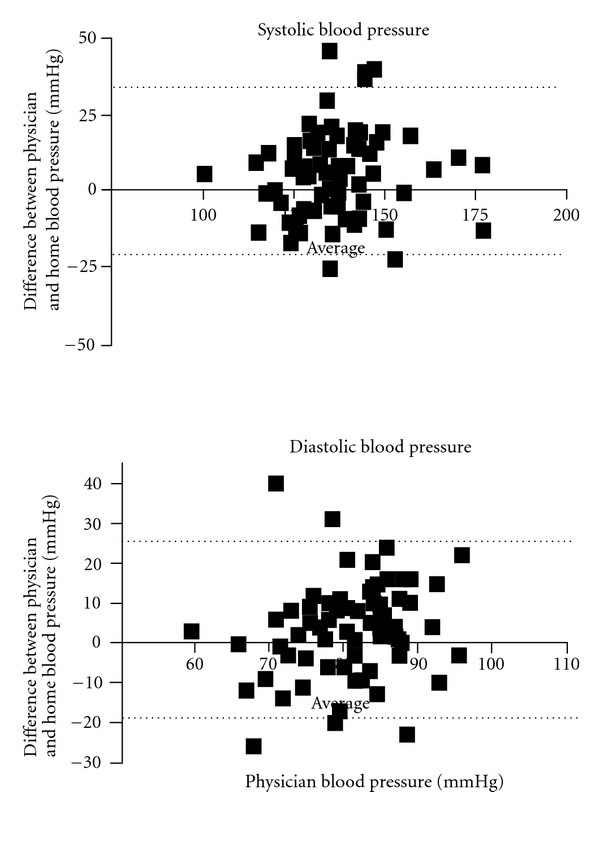
Bland-Altman figures of the physician and home blood pressure. Bland-Altman plots—the difference between the physician and home blood pressure—are plotted against the physician blood pressure; therefore, a positive difference suggests an overestimation by physician, whereas a negative difference suggests an underestimation. The solid lines represent the mean difference between physician and home blood pressure; the dashed lines represent the 95% lines of agreement. For systolic blood pressure, the values are bias: 5.86; SD of bias: 14.25; 95% limits of agreement (−22.08, 33.79). For diastolic blood pressure, the value is bias: 3.59; SD of bias: 11.80; 95% limits of agreement (−19.54, 26.72).

We then analyzed the BP values obtained with home BP measurement. The mean systolic and diastolic home BP values were, respectively,  133 ± 79  and 79 ± 8 mmHg. The office BP and the home BP were correlated (*r* ^2^ = 0.61; *P* < 0.01). However, using the definitions used (see Section 2), only 58% of the patients were classified in the same group with both methods (28% controlled and 30% uncontrolled hypertension). These figures are summarized in Table 2. Bland and Altman analysis of the data (clinical and HBP) contribute to show that discrepancies are evident in the whole range of analyzed pressures (Figure 1). Interestingly, we found that among 37 patients with controlled office BP, 16 patients (43.2%) exhibited home high (uncontrolled) blood pressure (masked hypertension).

### 3.3. Risk Factors for Blood Pressure Characteristics

We then compared the characteristics of patient according to the defined type of BP profile that they presented (Table 3). Uncontrolled AH was associated with an older age, a higher value of HbA1c, and more patients receiving antidiabetic treatment with poor control of the diabetes mellitus. As expected, more patients with uncontrolled and white-coat AH received antihypertensive treatment (resp., 96% and 87%), even though this difference is not statistically significant (*P* = 0.06). Furthermore, more than 40% of patients with uncontrolled AH received more than 2 drugs to try to control their blood pressure.

### 3.4. Renal Characteristics

We found no difference with regard of the presence of hypertension (uncontrolled, white-coat and masked) and the renal function assessed both by serum creatinine and glomerular filtration rate estimated by the MDRD formula.

However, patients with persistent uncontrolled arterial hypertension presented a significantly higher excretion of proteinuria when compared with all the other groups (Table 4). 26/51 and 14/23 of patients with controlled HT or noncontrolled HT, respectively, were under ARA2 or IEC (fisher test, nonsignificant). Among patients with noncontrolled HTN, frequency of proteinuria was not significantly different in patients with and without ARA2 or IEC treatment (0/9 versus 5/14, *P* > 0.05, Fisher test).

## 4. Discussion

Cardiovascular events are the first cause of death in RTR. Among cardiovascular risk factors, an elevated arterial blood pressure is recognized to be a strong predictor of graft function and patient death [1, 14–16]. Even though the prevalence of AH is high, the true prevalence is largely unknown since, to date, there is only few data regarding home BP which has become the gold standard for BP diagnosis and followup [17]. In fact Agarwal has showed that in chronic kidney disease, blood pressures obtained at home are a stronger predictor of end-stage renal disease (ESRD) or death compared to BPs obtained in the clinic. Systolic home BP is an independent predictor of ESRD [18].

We show here that, even though mean BP is relatively well controlled in our population, there is a large discrepancy between home BP and office BP (in up to 42% of patients) with both white-coat and masked AH. Since there is no good marker to discriminate between patients with concordant BP and the 2 previous populations, there is a need to perform both office and home BP.

The first question is the relative importance of home BP compared with office BP. First, we found a very good acceptability and compliance for home BP in the renal transplant population. Indeed, we found no phantom recordings. This may be due to the fact that patients were motivated as renal transplant recipients to assess their blood pressure with an extensive explanation of the positive impact both by doctors and the nurses. Stenehjem et al. have already showed that home BP was superior to office BP in estimating BP control in renal transplant patients with deteriorating graft function [12]. It can also be inferred from previous studies and the present results that the predictive power of home blood pressure is largely attributable to the fact that it has multiple measurements [17]. One potential interest may also be to estimate BP variability, which has been recently advocated to be of utmost importance [7, 8]. It has been also suggested that, not only the number of measurements but also such other factors as the lack of the white-coat effect, may be associated with the fact that predictive power of home blood pressure is superior to that of conventional casual blood pressure.

One interesting finding in our study is that patients with masked BP were also prone to present more often bad control of glycemia. This could be explained by poor compliance of both antihypertensive and diabetic drugs in the population.

One other potential advantage of home BP is the detection of both white-coat and masked hypertension. This latter may be important since it entailed an increased risk of ESRD, whereas the risk associated with white-coat hypertension was lower [19]. This may be related to the fact that masked hypertension by definition lasts a longer time than white-coat hypertension. The prevalence of masked hypertension in the general population is between 8 to 20% and can be up to 50% in treated hypertensive patients. Subjects with masked hypertension have a higher risk of cardiovascular accidents than normotensive subjects [19]. The variables that has been associated with a masked hypertension in nontransplant population are an office systolic BP higher than 130 mmHg, the male gender, patients age, more than two ongoing antihypertensive and a BMI higher than 25 [20]. Apart from patient age and the number of antihypertensive drugs, we did not find the same risk factors possibly because of the small sample size of our study. Of note, we found no difference with regard to the delay after transplantation.

In our study, we found that uncontrolled BP was associated with higher daily proteinuria excretion. Controlled AH was associated with the lowest value of protein excretion and both masked AH and white AH presented intermediate value. Studies with a higher number of patients and with a longer followup are needed to determine whether one or the other form of AH is associated with bad prognosis.

We also wanted to explore whether blood pressure assessment by a nurse would improve the accuracy of the diagnosis. Indeed, the comparison between the value of medical doctor and nurse BP measurements show that the white-coat effect is decreased by the nurse. Even though it is imperfect, we suggest that office BP should be better monitored by a nurse.

In conclusion, our study shows that in kidney transplants, home BP measurements is acceptable and may help to better define BP profile. There is a total of 42% discrepancy between office BP and home BP. The high incidence of both white-coat and masked hypertension suggests that the monitoring of RTR treated for hypertension must include home BP self-measurement. Our study demonstrates that proteinuria is higher with uncontrolled BP and show a tendency to be higher in masked hypertension. Followup of a larger population may allow demonstrating the negative effect of all profiles of hypertension. It also remains to be shown that the adaptation of treatment to the results of home BP self-measurement allows better cardiovascular prevention than adaptation of treatment to results of measurements in the physician's office.

### 4.1. Limitations

Our study has several limitations. First, it has been conducted only in a small pilot population. Second, even though it is unlikely, a systematic relationship between timing of antihypertensive drug ingestion and that of BP measurement could explain the better values of home over office BP measurement. Even though patients were asked to have their BP measured at a specific time interval after drug intake, we cannot eliminate this hypothesis since we did not record specifically these data. Another relationship could be proposed between the timing of blood analysis results announcement with the stress induced and the office BP measurement. In this regards, we could have compared the BP at the beginning and at the end of the office visit. Besides, one could argue that the BP threshold of home BP monitoring has not been established and is perhaps different from the general population or the patients under hemodialysis [21].

## Figures and Tables

**Table 1:** Demographic characteristics (mean ± SD, range).

	Total
Age (years)	48 ± 14 (24–79)
Body mass index (kg/m^2^)	24.8 ± 4.7 (18–44)
Gender (% males)	67
Posttransplantation delay (years) (median and range)	3.08 (1–31)
Office systolic blood pressure (mmHg)	139 ± 14 (103–181)
Office diastolic blood pressure (mmHg)	83 ± 10 (55–107)
Total cholesterol (mmol/L)	4.78 ± 0.97 (2.87–8)
LDL cholesterol (mmol/L)	2.49 ± 0.78 (1–4.33)
HDL cholesterol (mmol/L)	1.47 ± 1.09
Triglyceridemia (mmol/L)	1.68 ± 0.97 (0.5–7.3)
Diabetes (%)	6
Fasting glucose level (mmol/L)	5.07 ± 1.05
HbA1c (%)	5.87 ± 0.09
Current smoker (%)	5.1
Creatinine (*μ*mol/L)	137 ± 39 (72–302)
Proteinuria (g/L)	0.16 ± 0.27 (0–1.9)
Microalbuminuria (mg/j)	32.33 ± 70.3 (0–455)
Calcemia (mmol/L)	2.4 ± 0.18
Patients receiving anti-hypertensive drugs (%)	82.5
Number of antihypertensive drugs	1.5 ± 1

**Table 2:** Number of patients with controlled and uncontrolled home and office blood pressure (BP).

	Controlled home BP	Uncontrolled home BP
Controlled office BP	21	16
Uncontrolled office BP	16	22

**Table 3:** Patients characteristics classified by threshold of BP normality by measurement methods (AH: arterial hypertension).

	Uncontrolled AH	Masked AH	White-coat AH	Controlled AH	*P*
Age	55.30 ± 12.65	50.87 ± 15.08	50.93 ± 13.66	39.81 ± 10.82	0.002
Body mass index	26.61 ± 0.99	24.25 ± 1.18	24.13 ± 1.18	24.05 ± 1.03	0.23
Sex (male gender, %)	61%	50%	75%	81%	0.19
Delay	4,8 ± 4.4	5.12 ± 6.6	6.0 ± 6.1	7.3 ± 8.3	0.62
HbA1c	6.34 ± 0.18	5.91 ± 0.22	5.61 ± 0.22	5.59 ± 0.65	0.03
HbA1c > 6.5% in treated patients	30%	6%	0%	5%	0.009
Total cholesterol	4.96 ± 1.17	4.86 ± 1.10	4.78 ± 0.64	4.58 ± 0.79	0.61
Triglycerides	1.64 ± 0.62	1.92 ± 0.97	1.94 ± 1.62	1.46 ± 0.63	0.39
LDL cholesterol	2.53 ± 0.60	2.49 ± 0.82	2.63 ± 0.42	2.36 ± 0.85	0.71
C2 cyclosporine	796 ± 146	673 ± 260	586 ± 24	580 ± 213	0.28
C0 tacrolimus	7.3 ± 0.6	8.4 ± 0.7	7.6 ± 0.7	7.7 ± 0.6	0.69
BP treatment (Yes)	96%	80%	87%	67%	0.06

**Table 4:** Renal associations with hypertension.

	Uncontrolled hypertension	Masked hypertension	White-coat hypertension	Controlled hypertension	*P*
Serum creatinine	138.3 ± 20.5	131.3 ± 32.4	125.9 ± 38.1	142.0 ± 41.9	0.48
eGFR	42.8 ± 9.4	47.4 ± 15.3	53.0 ± 14.9	49.8 ± 16.3	0.14
Proteinuria	0.29 ± 0.44	0.14 ± 0.18	0.11 ± 0.13	0.07 ± 0.09	0.05

## Abbreviations

BP: Blood pressure
HBP: Home blood pressure
HBPM: Home blood pressure measurement
OBP: Office blood pressure
OBPM: Office blood pressure measurement
RB: Renal biopsy.
